# Induction of Hemeoxygenase-1 Reduces Renal Oxidative Stress and Inflammation in Diabetic Spontaneously Hypertensive Rats

**DOI:** 10.1155/2012/957235

**Published:** 2012-02-26

**Authors:** Ahmed A. Elmarakby, Jessica Faulkner, Babak Baban, Jennifer C. Sullivan

**Affiliations:** ^1^Department of Oral Biology, Georgia Health Sciences University, Augusta, GA 30912, USA; ^2^Department of Medicine, Georgia Health Sciences University, Augusta, GA 30912, USA

## Abstract

The renoprotective mechanisms of hemeoxygenase-1 (HO-1) in diabetic nephropathy remain to be investigated. We hypothesize that HO-1 protects the kidney from diabetic insult via lowering renal oxidative stress and inflammation. We used control and diabetic SHR with or without HO-1 inducer cobalt protoporphyrin (CoPP) treatment for 6 weeks. Urinary albumin excretion levels were significantly elevated in diabetic SHR compared to control and CoPP significantly attenuated albumin excretion. Immuno-histochemical analysis revealed an elevation in TGF-*β* staining together with increased urinary collagen excretion in diabetic versus control SHR, both of which were reduced with CoPP treatment. Renal oxidative stress markers were greater in diabetic SHR and reduced with CoPP treatment. The increase in renal oxidative stress was associated with an elevation in renal inflammation in diabetic SHR. CoPP treatment also significantly attenuated the markers of renal inflammation in diabetic SHR. In vitro inhibition of HO with stannous mesoporphyrin (SnMP) increased glomerular NADPH oxidase activity and inflammation and blocked the anti-oxidant and anti-inflammatory effects of CoPP. These data suggest that the reduction of renal injury in diabetic SHR upon induction of HO-1 are associated with decreased renal oxidative stress and inflammation, implicating the role of HO-1 induction as a future treatment of diabetic nephropathy.

## 1. Introduction

The incidence of diabetes mellitus has dramatically increased worldwide [1, 2]. One of the major complications of diabetes is the progression of renal injury, affecting approximately 35% of type 1 and type 2 diabetic patients, which often leads to end-stage renal disease. Diabetes is often associated with an elevation in blood pressure which is known to worsen renal function [3–5]. Accordingly, we induced diabetes in spontaneously hypertensive rats (SHR) in the current study as a genetic model of essential hypertension to address the effects of diabetes on a hypertensive background.

 Increased oxidative stress has been implicated in the pathogenesis of diabetes and hypertension [6, 7]. NADPH oxidase, the major source of superoxide production in the vasculature, is known to activate numerous inflammatory cytokines [8]. NADPH oxidase has been shown to be activated in the kidney of diabetic animal models, with enhanced expression in the glomerulus and distal tubules [9–11]. NADPH oxidase-derived reactive oxygen species increase renal hypertrophy and fibronectin expression in streptozotocin-induced type 1 diabetic rats [11, 12] as well as exacerbate the damage in glomerular basement membrane and slit diaphragm [10, 13]. Collectively, these data suggest that NADPH oxidase-derived superoxide contributes to the progression of diabetic-induced renal injury.

 Clinically, inflammatory processes in the kidney also contribute to the progression of nephropathy in patients with type 1 diabetes and in diabetic animal models [14–19]. Diabetic renal injury is an inflammatory disease characterized by monocyte infiltration at every stage of the disease progression with chemokines driving the recruitment of inflammatory cells into renal compartments [15, 18]. Kidney of diabetic humans and experimental animal models both show increased macrophage infiltration and overproduction of leukocyte adhesion molecules [14–19]. Activated inflammatory cells further exacerbate cytokine release leading to enhanced fibrosis, matrix deposition, and progressive renal injury. Moreover, oxidative stress has been demonstrated to modulate expression of many inflammatory genes in diabetes, including cell adhesion molecules (CAMs) and monocyte chemoattractant protein (MCP-1). Taken together, these data support a role of immune response in the progression of diabetic renal injury [11, 20].

Heme catabolism is primarily driven by hemeoxygenase (HO) generating biliverdin, iron, and carbon monoxide [21, 22]. There are two isoenzymes of HO: inducible HO-1 and constitutive HO-2 which accounts for most HO activity in the normal state [21, 22]. Studies have shown that HO-1 is upregulated in response to oxidative stress, ischemia, and inflammation [21, 22]. Induction of HO-1 also reduces blood pressure and inflammation in experimental models of diabetes and hypertension suggesting that HO-1 induction may protect the diabetic kidney via inhibition of oxidative stress and inflammation [23–26].

Previous studies have suggested a role for hyperglycemia in increasing oxidative stress and inflammation in diabetic animal models [27, 28]; however, most of the studied diabetic animal models remained normotensive. Because diabetic nephropathy is characterized by increased albuminuria with an elevation in blood pressure and decline in renal function, the coexistence of hypertension and diabetes in the current animal model is expected to worsen the degree of renal injury and more accurately reflect the clinical picture of diabetic nephropathy. The current study tests the hypothesis that HO-1 induction-mediated decreases in renal injury are associated with decreases in renal oxidative stress and inflammation in diabetic SHR.

## 2. Materials and Methods

 All procedures with animals were performed in accordance with the Public Health Service Guide for the Care and Use of Laboratory Animals and Georgia Health Sciences University guidelines. Eleven-week-old male SHR (Charles River, MA) were used to induce diabetes by a single injection of streptozotocin (Sigma, MO; 65 mg/kg i.v dissolved in 0.1 M citrate buffer) and control SHR only received 0.1 M citrate buffer injection. The blood glucose levels of these rats were maintained within 400–500 mg/dL via the use of sustained release insulin implants (s.c, Lanshin, Canada), and blood glucose levels were tested weekly using a glucometer. Normal control and diabetic SHR rats were randomized to receive either vehicle (0.1 M NaOH, pH 8.3) or the HO-1 inducer cobalt protoporphyrin (CoPP, 5.0 mg/100 g body weight s.c) weekly for six weeks after induction of diabetes (*n* = 8/group). Systolic blood pressure was recorded weekly using the tail cuff method (IITC Life Science, Woodland Hills, CA) [25]. Rats were placed in metabolic cages (Nalgene Corp. Rochester, NY) for 24-hour urine collection at the end of the experiment. Urinary creatinine (Cayman Chemical, Ann Arbor, MI), albumin, and collagen (Exocell, Philadelphia, PA) excretion levels were determined as indices of renal injury. Urinary thiobarbituric acid reactive substances (TBARs, Cayman Chemical, Ann Arbor, MI), and 8-hydroxy deoxyguanosine (*8-OHdG, *Northwest, WA) excretion levels were assessed as markers of oxidative stress.

### 2.1. Homogenization of the Renal Cortex for Protein Expression Using Western Blotting Analysis

 Renal cortical samples were homogenized in RIPA buffer supplemented with inhibitors for proteases and phosphatases as previously described [25]. Protein concentrations were determined by Bradford assay (Bio-Rad, Hercules, CA). Cortical samples were separated by SDS-PAGE as previously described [25]. Gels were then transferred onto nitrocellulose membranes. The primary antibodies used were: rabbit HO-1, HO-2 (EMD Biosciences, San Diego, CA), and mouse *β*-actin (Sigma, St. Louis, MO). These antibodies were detected with a horseradish peroxidase-conjugated secondary antibody and ECL chemiluminescence (Amersham BioSciences, Buckinghamshire, UK). Intensity of immunoreactivity was measured by densitometry, and *β*-actin was used to verify equal loading of protein.

### 2.2. Renal NADPH Oxidase Activity

NADPH activity was measured in cortical samples by lucigenin chemiluminescence using 35 *μ*g protein in the presence of NADPH (100 *μ*M) and lucigenin (5 *μ*M) as previously described [29] and average sample counts (cpm) were normalized to *μ*g protein.

### 2.3. Renal MCP-1, HO-1, and sICAM-1 Assays

HO-1 activity was measured in renal cortical samples using a commercially available ELISA according to manufacturer's instructions (Enzo Life Sciences Inc., Farmingdale, NY). Renal cortical MCP-1 levels were assessed using a commercially available ELISA according to manufacturer's instructions (BD Biosciences, Bedford, MA). Renal soluble ICAM-1 levels (sICAM-1) were also determined using a commercially available ELISA according to manufacturer's instructions (R&D Systems, Minneapolis, MN).

### 2.4. Renal Histopathology

In a separate set of rats (*n* = 5/group), kidneys were perfused with 10% formalin solution and were then paraffin embedded and cut into 4- to 5-*μ*m sections. Kidney sections were used for immunohistochemical evaluation of CD68 to assess monocyte/macrophage infiltration (ED-1 staining) and CD3 to assess T-cell infiltration as previously described [30]. Ten microscopic images of the kidney cortex per rat were randomly taken at ×200 magnification, and CD68-positive and CD3-positive cells were counted by a blinded reviewer experienced in analysis. The number of positive cells per millimeter squared was calculated and averaged for each group. Additional kidney sections were immunohistochemically stained with TGF-*β* antibodies (Santa Cruz Biotechnology, Santa Cruz, CA), and staining intensity was evaluated at ×200 and ×400 magnification power, respectively. Masson's trichrome staining of kidney sections was also used to assess the amount of collagen deposition, ×200 magnification. 

### 2.5. Isolation of Glomeruli

 Glomeruli were isolated as previously described [39] by a gradual sieving technique from control and diabetic SHR and incubated for 2 hours at 37°C with the HO inhibitor stannous mesoporphyrin (SnMP, 20 mM), CoPP (10 mM), or both SnMP and CoPP (*n* = 4/group). Glomerular NADPH activity was determined by lucigenin method, and glomerular P-ERK/ERK ratio was also assessed by Western blotting using antibodies from cell-signaling technology (Beverly, MA). 

### 2.6. Data Analyses

Statistical analyses were performed using Prism software (GraphPad, San Diego, CA, USA). Data were reported as means ± SEM and were analyzed using one-way analysis of variance (ANOVA) followed by Tukey's post-hoc test (*P* < 0.05 was considered significant).

## 3. Results

**Figure 1:**
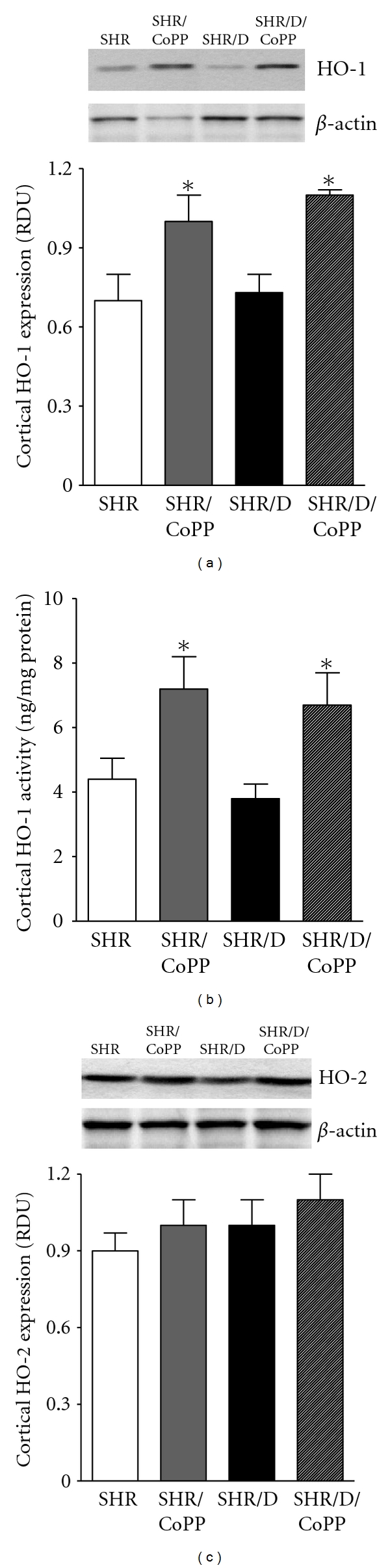
Renal cortical HO-1 expression relative to *β*-actin (a), HO-1 activity (b), and HO-2 expression relative to *β*-actin (c) in control and diabetic (D) SHR with or without CoPP treatment (*n* = 6, *indicates significant difference from control SHR and ^#^indicates significant difference from diabetic SHR).

As shown in Figures 1(a) and 1(b), induction of diabetes with streptozotocin did not significantly change renal HO-1 activity or expression in SHR. However, CoPP treatment significantly elevated renal HO-1 expression and activity in both control and diabetic SHR (*P* < 0.05). There was no difference in renal HO-2 expression among all rat groups (Figure 1(c)). Induction of diabetes did not significantly change systolic blood pressure in SHR (209 ± 4 versus 200 ± 4 mmHg) although blood glucose levels were significantly elevated compared to control SHR (507 ± 43 versus 205 ± 15 mg/dL). CoPP treatment reduced blood pressure (187 ± 2 mmHg) and blood glucose (425 ± 47 mg/dL) in diabetic SHR; however, blood glucose and blood pressure remained significantly higher than control SHR.

### 3.1. Renal Injury

**Figure 2:**
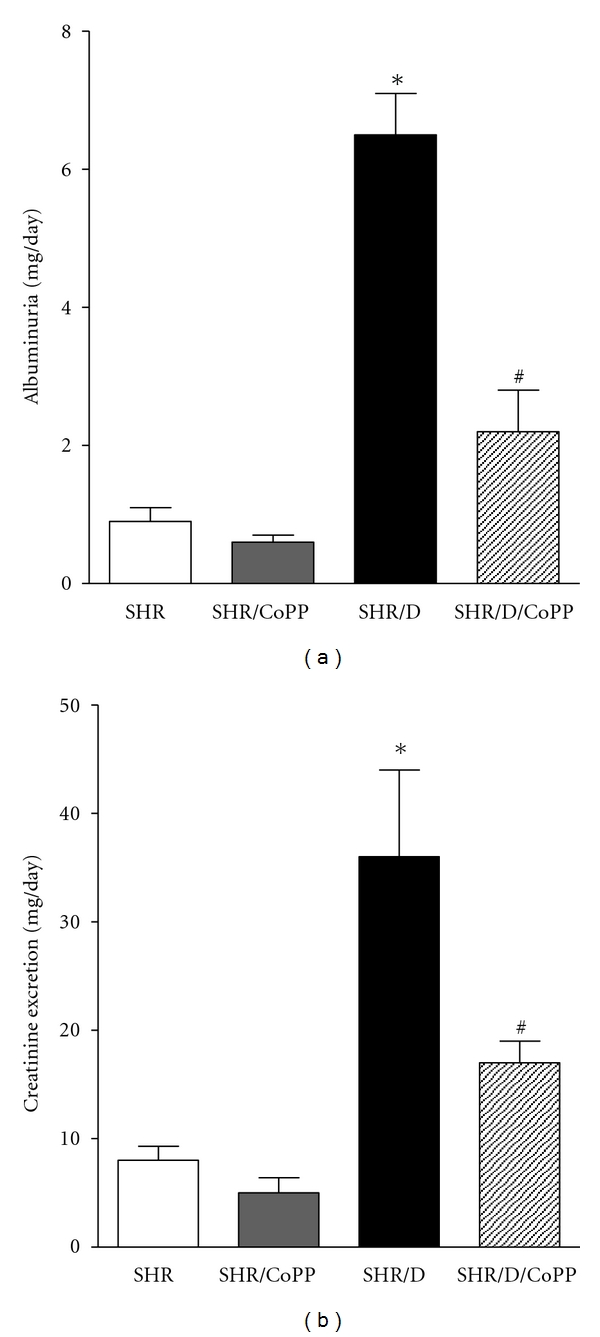
Urinary albuminuria (a) and creatinine excretion (b) in control and diabetic SHR with or without CoPP (*n* = 8, *indicates significant difference from control SHR and ^#^indicates significant difference from diabetic SHR).

We assessed urinary albumin and creatinine excretion levels as markers of renal injury. Control SHR had a significantly higher level of albuminuria than normotensive WKY (1.0 ± 0.2 versus 0.35 ± 0.05 mg/day, *P* < 0.05). As shown in Figure 2(a), diabetic SHR exhibited a significant increase in albuminuria after 6 weeks of induction of diabetes compared to control SHR (6.5 ± 0.6 versus 1.0 ± 0.2 mg/day, resp., *P* < 0.05). CoPP treatment lowered albuminuria in control SHR (0.6 ± 0.1 mg/day) and significantly attenuated the elevation in albuminuria in diabetic SHR (2.2 ± 0.6, *P* < 0.05). Similarly, creatinine excretion was significantly elevated in diabetic SHR compared to control and was reduced with CoPP treatment (Figure 2(b)).

**Figure 3:**
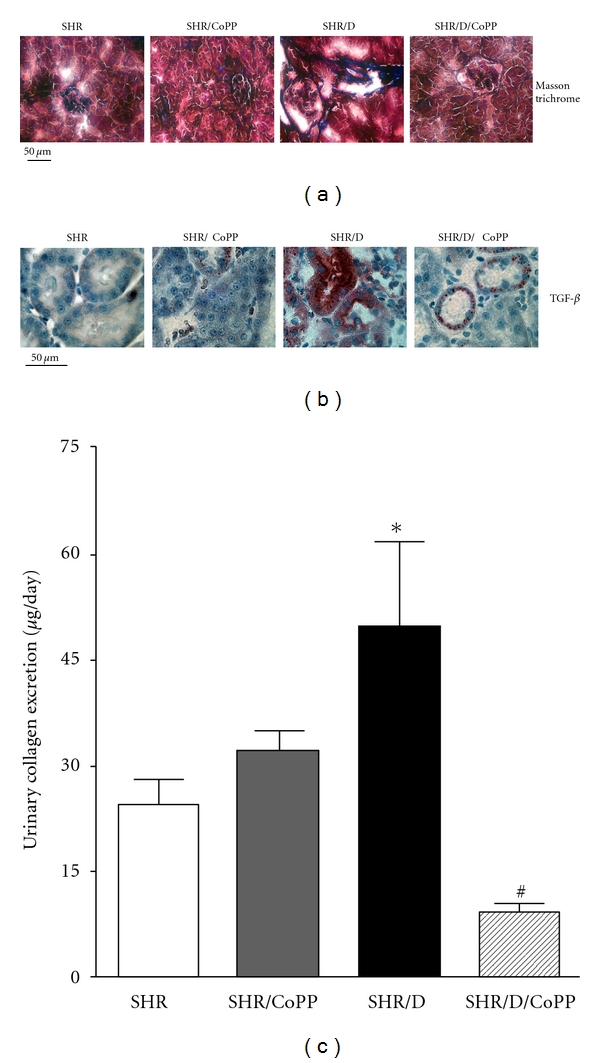
Representative images of Masson's trichrome staining (a) and immunohistochemical staining of TGF-*β* (b) in kidney sections from control and diabetic SHR with or without CoPP treatment (*n* = 5). (c) is urinary collagen excretion in control and diabetic SHR with or without CoPP treatment (*n* = 8, *indicates significant difference from control SHR and ^#^indicates significant difference from diabetic SHR).

The progression of renal injury in diabetic SHR was associated with renal vascular remodeling and increased extracellular matrix deposition and fibrosis as manifested by greater collagen deposition (blue staining, Figure 3(a)) and enhanced TGF-*β* levels (red staining, Figure 3(b)) in diabetic SHR. The increase in collagen deposition was also associated with an elevation in urinary collagen excretion in diabetic SHR compared to control SHR (Figure 3(c)). Induction of HO-1 with CoPP reduced collagen deposition and TGF-*β* staining and significantly lowered urinary collagen excretion in diabetic SHR (Figure 3).

### 3.2. NADPH Oxidase and Oxidative Stress

**Figure 4:**
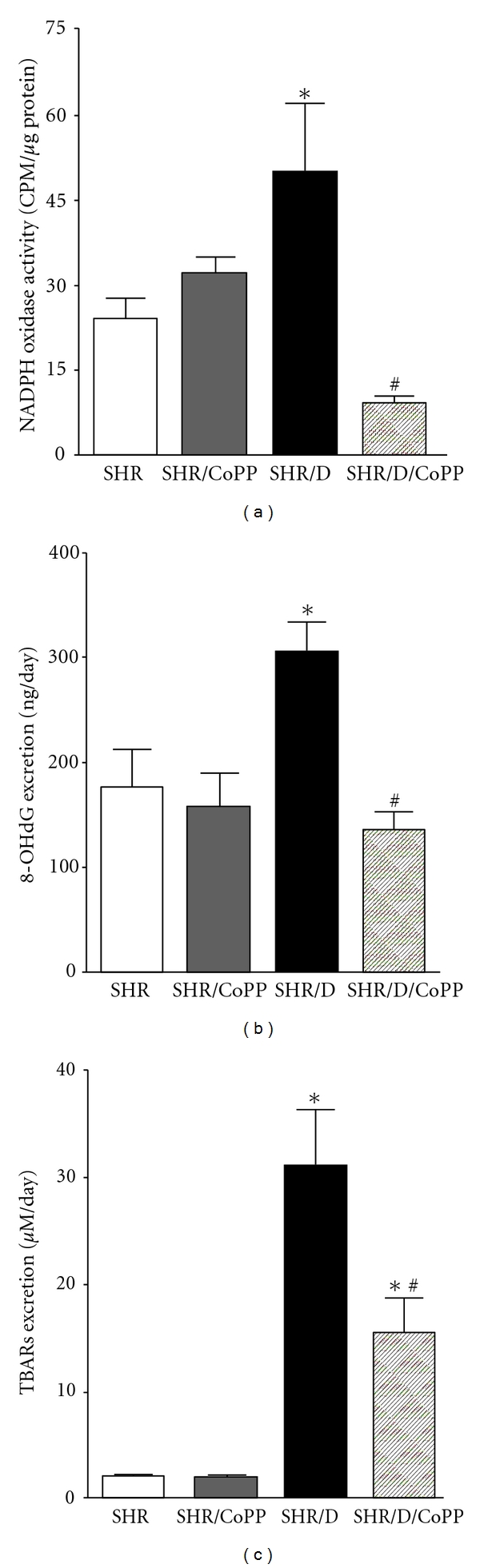
Cortical NADPH oxidase activity (a), urinary thiobarbituric acid reactive substances (TBARs) and urinary *8-hydroxy deoxyguanosine (8-OHdG)* excretion levels (b) and (c), respectively, in control and diabetic SHR with or without CoPP treatment (*n* = 8, *indicates significant difference from control SHR, ^#^indicates significant difference from diabetic SHR).

Oxidative stress has been shown to play a role in the pathogenesis of diabetic-induced renal injury, and NADPH oxidase is the main source of superoxide production in diabetes [10, 11]. Consistent with these observations, renal cortical NADPH oxidase activity was significantly elevated in diabetic SHR compared to control SHR (Figure 4(a)). The increase in NADPH oxidase activity was also associated with elevation in the oxidative stress markers TBARs and *8-OHdG *excretion levels in diabetic versus control SHR (Figures 4(b) and 4(c)). Induction of HO-1 with CoPP inhibited NADPH oxidase activity and reduced excretion levels of oxidative stress markers in diabetic SHR (Figure 4). Plasma TBARs were also elevated in diabetic versus control SHR (39 ± 8 versus 26 ± 4 *μ*M) and were reduced with CoPP treatment in diabetic and control SHR (26 ± 5 & 13 ± 4 *μ*M, resp.) suggesting that induction of HO-1 lowers renal as well as systemic oxidative stress levels.

### 3.3. Renal Inflammation

**Figure 5:**
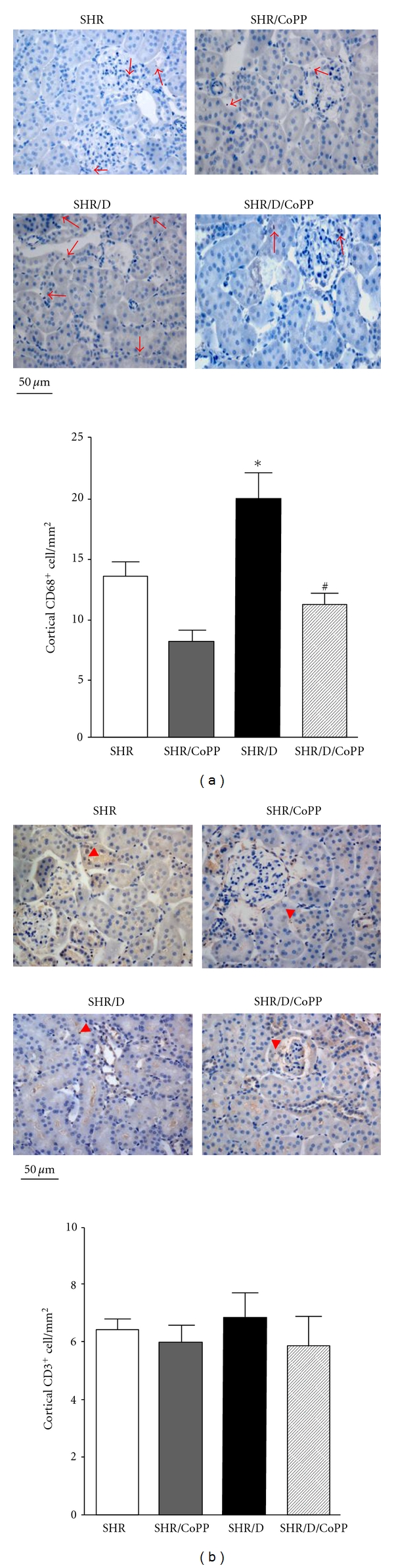
Representative images and average number of CD68-positive and CD3-positive cells (C) to assess monocytes/macrophages and T-cell infiltration, respectively, per 1 mm^2^ in the kidney cortex of control and diabetic SHR with or without CoPP treatment (*n* = 5, *indicates significant difference from control SHR, ^#^indicates significant difference from diabetic SHR).

Because diabetic renal injury is characterized by leukocyte infiltration at every stage of the disease progression [14, 18], kidneys were processed for immunohistochemical quantification of macrophage (CD68) and T-cell (CD3) infiltration. Macrophage infiltration was significantly greater in diabetic versus control SHR and induction of HO-1 with CoPP significantly attenuated the increase in macrophage infiltration in diabetic SHR (Figure 5(a)). There was no difference in T-cell infiltration between all groups (Figure 5(b)).

**Figure 6:**
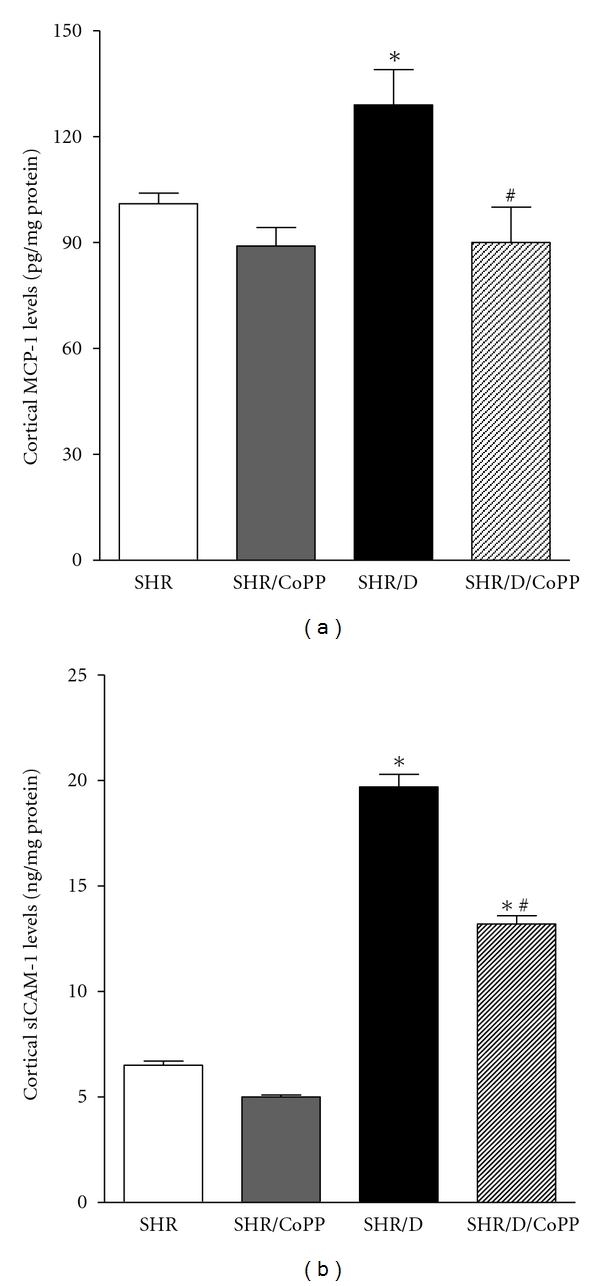
Renal cortical MCP-1 (a) and sICAM-1 (b) levels in control and diabetic SHR with or without CoPP treatment (*n* = 8, *indicates significant difference from control SHR, ^#^indicates significant difference from diabetic SHR).

We have recently shown that the NF*κ*B inflammatory signaling pathway plays a crucial role in the progression of diabetic renal injury via the activation of proinflammatory molecules such as MCP-1 [31]. Consistent with this observation, renal cortical MCP-1 levels were significantly elevated in diabetic SHR compared to control SHR (129 ± 10 versus 101 ± 3 pg/mg protein, *P* < 0.05), and levels were reduced with CoPP treatment in diabetic SHR (90 ± 10 pg/mg protein, Figure 6(a)). Similarly, renal sICAM-1 levels were significantly elevated in diabetic SHR compared to control SHR and reduced with CoPP treatment (Figure 6(b)).

### 3.4. *In Vitro* Inhibition of HO in Isolated Glomeruli

**Figure 7:**
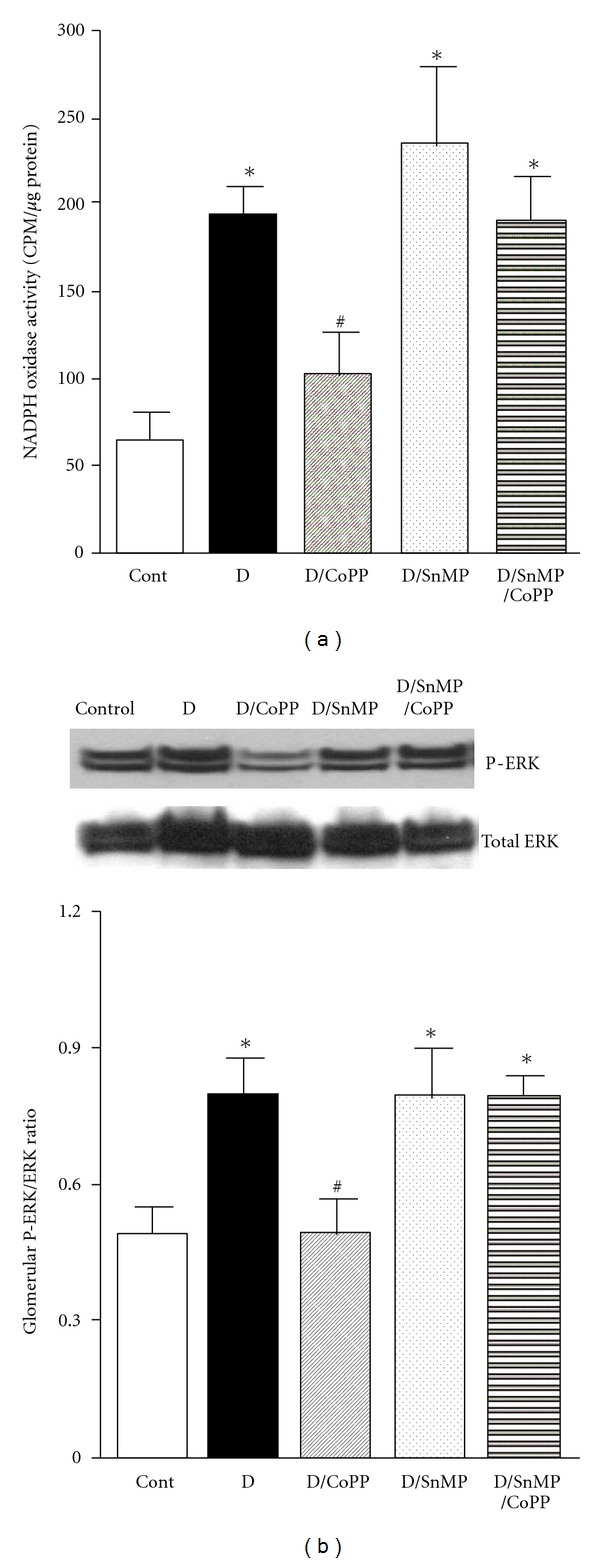
NADPH oxidase activity (a) and P-ERK/ERK ratio (b) in glomeruli isolated from control and diabetic SHR and incubated with or without CoPP and/or SnMP for 2 hours at 37°C (*n* = 4, Cont is an abbreviation for control SHR and D is an abbreviation for diabetic SHR, *indicates significant difference from control SHR, ^#^indicates significant difference from diabetic SHR).

Glomerular NADPH oxidase was significantly elevated in diabetic versus control SHR and incubation of isolated glomeruli from diabetic SHR with CoPP reduced NADPH oxidase activity (Figure 7(a)). Treatment of isolated glomeruli from diabetic SHR with the HO inhibitor SnMP further increased NADPH oxidase and prevented the ability of CoPP to reduce NADPH oxidase activity (Figure 7(a)). Previous studies demonstrated that MAPK activation is involved in the secretion of proinflammatory cytokines [32, 33] and increased ERK phoshorylation could be an indicative of renal inflammation during diabetes [34]. In our study, CoPP treatment significantly inhibited hyperglycemia-induced ERK phosphorylation in glomeruli isolated from diabetic SHR, and this effect was also prevented with SnMP treatment (Figure 7(b)).

## 4. Discussion

The current study provides evidence that HO-1 induction mitigates renal injury and inflammation in type 1 diabetic SHR as a model in which diabetes coexists with hypertension to exaggerate the progression of renal injury. Induction of HO-1 attenuated the elevation in albuminuria and creatinine excretion in diabetic SHR and decreased renal fibrosis and extracellular matrix deposition in the kidney of diabetic SHR as evidence by decreased kidney TGF-*β* and collagen and decreased collagen excretion in diabetic SHR. It is now widely acceptable that oxidative stress and inflammatory cytokines play a crucial role in the progression of diabetic renal injury. Interestingly, HO-1 induction inhibited NADPH oxidase activation and reduced markers of oxidative stress in diabetic SHR. HO-1 induction also reduced kidney macrophage infiltration and attenuated renal MCP-1 and sICAM-1 levels in diabetic SHR. Inhibition of HO with SnMP negated the protective effect of CoPP on NADPH oxidase activation and ERK phosphorylation in isolated glomeruli from diabetic SHR. These findings suggest that induction of HO-1 could function to protect the kidney from diabetes-induced renal injury. We postulate that the renoprotective effects of HO-1 induction could be linked to inhibition of renal NADPH oxidase-derived oxidative stress and inflammation in diabetic SHR.

The potential renoprotective mechanisms of HO-1 induction remain to be explored. Induction of HO-1 has been shown to decrease blood pressure in experimental hypertensive and diabetic animal models including SHR [24–26]. Consistent with the previous findings, HO-1 induction lowered blood pressure in control and diabetic SHR; however, this is unlikely to be the sole renoprotective mechanism as the blood pressure of CoPP-treated SHR remained very high. Besides, a blood pressure lowering effect, induction of HO-1 with CoPP has also previously been shown to reduce fasting blood glucose and plasma levels of inflammatory cytokines in obese male and female mice suggesting the potential beneficial effects of HO-1 in treating not only hypertension, but also the metabolic consequences of obesity such as insulin resistance and dyslipidemia [23]. In support of this hypothesis, HO-1 upregulation has been shown to improve insulin sensitivity and glucose metabolism in SHR [35] which could be another potential renoprotective mechanism in diabetes. Consistent with these findings, induction of HO-1 with CoPP in this current study decreased blood glucose levels in control and diabetic SHR; however, it is unlikely to be the only mechanism of CoPP-induced kidney protection as blood glucose levels in CoPP-treated diabetic SHR remained significantly higher than control SHR. Overall, the hypotensive and hypoglycemic effects of HO-1 induction could contribute, in part, to the renal protection against diabetic insult.

HO-1 has been implicated in the modulation of renal injury in hypertensive animal models. For example, induction of HO-1 with hemin has also been shown by others to attenuate proteinuria and tubular atrophy in salt-sensitive angiotensin II hypertension [36]. Hemin also ameliorated renal injury in angiotensin II hypertension as it prevented the decrease in glomerular filtration rate and reduced proteinuria [37]. In SHR, we have recently shown that induction of HO-1 with CoPP reduced proteinuria when compared to Wistar Kyoto rats (WKY), whereas inhibition of HO with stannous mesoporphyrin further increased blood pressure and proteinuria and blocked the ability of CoPP to reduce blood pressure and proteinuria in SHR. In diabetes, HO-1 could also play a role in preserving renal function and morphology. For example, induction of diabetes with streptozotocin produced a marked degree of renal impairment in HO-2 knockout mice compared to control [38]. Furthermore, induction of HO-1 with CoPP prevented the elevation in plasma creatinine levels and acute tubular damage in diabetic HO-2 knockout mice whereas inhibition of HO with tin mesoporphyrin exacerbated the increase in plasma creatinine and tubular damage in diabetic HO-2 knockout mice [38]. Consistent with these observations, the coexistence of hypertension and diabetes in diabetic SHR exaggerated the degree of renal injury as manifested by increased albumin and creatinine excretion and induction of HO-1 with CoPP reduced these changes.

Although induction of diabetes with streptozotocin in Sprague Dawley rats does not have extensive fibrosis as detected by histological staining [28], Saleh et al. recently demonstrated that glomerular TGF-*β*, an early marker of fibrosis increased in streptozotocin-induced diabetic rats [39]. Others have shown that that overexpression of glomerular TGF-*β*1 in diabetes contributes to glomerular basement membrane thickening and fibrosis [40], and inhibition of TGF-*β* prevents kidney fibrosis in experimental diabetes [41] suggesting an important role of TGF-*β* in the progression of kidney fibrosis during diabetes. Previous studies have demonstrated that induction of HO-1 with hemin reduced the overexpression of osteopontin and TGF-*β*, the hallmarks of tubulointerstitial injury in salt-sensitive angiotensin II hypertension suggesting a role of HO-1 induction against renal fibrosis in hypertension [36]. In our study, the coexistence of hypertension and diabetes in diabetic SHR also exaggerated the degree of renal fibrosis as reflected by increased renal TGF-*β*1 staining, collagen IV deposition, and urinary collagen excretion compared to control SHR. The incidence of renal fibrosis was significantly reduced with CoPP treatment suggesting that HO-1 induction protects the kidney from diabetic-induced renal damage and fibrosis.

Increased oxidative stress is involved in the development of diabetic renal injury, and overexpression of HO-1 has previously been shown to decrease oxidative stress in diabetic animals [38, 42, 43]. Thus, the induction of HO-1 could provide cellular protection against oxidative insult during diabetes. In SHR, elevated oxidative stress and inflammatory markers not only accentuate oxidative damage but also impair the insulin signaling [35]. In streptozotocin-induced diabetic SHR, upregulation of HO-1 with stannous chloride was associated with a concomitant decrease in renal superoxide levels [24]. HO-1 upregulation by CoPP attenuated diabetic injury in nonobese diabetic (NOD) mice, an animal model for type 1 diabetes, and this was associated with decreases in blood glucose and pancreatic superoxide [44]. HO-1 induction also reduces aortic superoxide generation via decreased NADPH oxidase activation in apolipoprotein E-deficient mice [45]. Consistent with previous reports, our study showed that induction of HO-1 decreased renal cortical NADPH oxidase activity and urinary TBARs and 8-OHdG excretion levels in diabetic SHR. These data support the conclusion that HO-1 induction inhibits renal NADPH oxidase activity and reduces markers of oxidative stress, which could be a mechanism to protect the kidney against diabetic-induced renal injury.

Clinically, inflammatory processes contribute to the progression of renal injury in patients with type 1 diabetes [14, 46]. MCP-1 and ICAM-1 have been identified as key players in monocyte/macrophage infiltration and leukocyte adhesion in diabetic animal models [47, 48]. Many factors contribute to the increase in ICAM-1 production during diabetes including hyperglycemia, shear stress, advanced glycation end products, and oxidative stress [46]. Blocking ICAM-1 signaling abrogated the infiltration of macrophages in kidneys from diabetic rats and decreased glomerular hypertrophy and interstitial fibrosis in ICAM-1-deficient mice [48, 49] indicating a potential role of ICAM-1 in the progression of renal injury during diabetes. MCP-1 is also a potent chemoattractant for monocytes/macrophages and increased MCP-1 production was associated with macrophage infiltration in the kidney of diabetic patients [50]. MCP-1 is involved in the progression of kidney injury in response to many factors such as high glucose, oxidative stress, and interleukin-1 [27, 51]. MCP-1-deficiency or blocking MCP-1 receptor in mice reduced kidney macrophage accumulation and decreased renal injury in diabetes [16, 52] underscoring the importance of this pathway in the pathogenesis of diabetic renal injury. We have previously shown that induction of HO-1 with CoPP decreased MCP-1 excretion, whereas inhibition of HO with stannous mesoporphyrin blocked the ability of CoPP to decrease MCP-1 in SHR [25]. In our current study, induction of HO-1 with CoPP decreased the activation of renal MCP-1 and sICAM-1 together with decreased kidney macrophage, but not T-cell infiltration in diabetic SHR. Similarly, isolated glomeruli from diabetic SHR had a significant elevation in NADPH oxidase activity and ERK phosphorylation, and these effects were reduced with CoPP treatment and prevented by HO inhibition with SnMP. These data suggest that HO-1 upregulation reduces renal inflammation in diabetic SHR which could be an additional mechanism protecting the kidneys from diabetic insults.

In summary, HO-1 induction improves renal damage and decreases fibrosis in diabetic SHR. Based on data in the literature and our own studies, we postulate that hyperglycemia increases NADPH oxidase-induced oxidative stress which enhances the activation of proinflammatory cytokines stimulating immune cell infiltration and further increasing oxidative stress thereby exacerbating renal injury and fibrosis. The study highlights the potential therapeutic benefit of HO-1 induction to protect the kidney from diabetic renal injury via antioxidant and anti-inflammatory properties.
